# Utilization of antenatal care among adolescent and young mothers in Ghana; analysis of the 2017/2018 multiple indicator cluster survey

**DOI:** 10.1186/s12884-022-04872-z

**Published:** 2022-07-05

**Authors:** Emmanuel Anongeba Anaba, Stanley Kofi Alor, Caroline Dinam Badzi

**Affiliations:** 1 Depatment of Population, Family and Reproductive Health, School of Public Health, University of Ghana, Legon, Accra, Ghana; 2 Department of Social and Behavioural Sciences, School of Public Health, University of Ghana, Legon, Accra, Ghana; 3 Nursing and Midwifery Training College, 37 Military Hospital, Neghelli Barracks, Accra, Ghana; 4 Department of Maternal and Child Health, School of Nursing and Midwifery, University of Ghana, Legon, Accra, Ghana

**Keywords:** Adolescent and young mothers, Antenatal care, Utilization, Ghana, Multiple Indicator cluster survey

## Abstract

**Background:**

Complications during pregnancy and childbirth are the leading cause of death among adolescent girls. In Ghana, the prevalence of adolescent pregnancy remains high. Yet, little is known about ANC utilization among adolescent and young mothers. This study aimed to assess the prevalence of obtaining 4 or more ANC visits and associated factors among adolescent and young mothers.

**Methods:**

We analysed secondary data from the sixth round of the Ghana Multiple Indicator Cluster Survey. A total of 947 adolescent and young mothers were included in this study. Data were analysed using STATA/SE, version 16, employing descriptive statistics and Binary Logistic Regression.

**Results:**

It was found that majority of the participants were aged 20-24 years (70%), married/in union (61%) and non-insured (64%). The prevalence of obtaining 4 or more ANC visits was 84%. Adolescent and young mothers with junior high school education, in the second wealth quintile, exposed to the internet, and resided in the Upper East region had a higher likelihood of obtaining 4 or more ANC visits (*p* < 0.05).

**Conclusions:**

This study demonstrated that optimal ANC utilization among adolescent and young mothers were determined by socio-economic factors. Going forward, maternal healthcare interventions must prioritize adolescent and young mothers from poor socio-economic backgrounds.

## Background

Although much progress has been made in the last two decades from maternal healthcare interventions across the world, the maternal mortality ratio is still high. Globally, 295,000 women died of pregnancy and childbirth-related complications in 2017 [1]. The majority (94%) of these deaths occurred in low-resource settings [1]. Therefore, Target 3.1 of the Sustainable Development Goals seeks to reduce the global maternal mortality ratio to less than 70 per 100,000 live births by 2030 [2, 3]. Adolescent pregnancy is a risk factor for maternal mortality. Adolescent girls face a higher risk of complications, such as eclampsia, puerperal endometritis, systemic infections and death [1, 4]. Complications during pregnancy and childbirth are the leading cause of death among adolescent girls aged 15-19 years globally [5]. About 99% of maternal deaths worldwide occur in developing countries with the majority occurring in sub-Saharan Africa [6].

Antenatal care (ANC) services present a golden opportunity to help reduce maternal mortality. Although many maternal complications are difficult to detect during ANC visits, some, such as hypertensive disorders can be identified and proactively managed through antenatal care visits [4]. Pregnant women have access to skilled healthcare and early detection of danger signs in pregnancy during ANC visits. There is a positive relationship between ANC utilization and positive pregnancy outcomes [7]. Also, ANC enables expectant mothers to learn about signs of obstetric complications and the essence of accessing skilled delivery services [7]. ANC can help reduce perinatal and new-born morbidity and mortality [8–11].

Despite the benefits associated with ANC services, utilization remains low worldwide [1], especially among young mothers (15-24 years) [12–15]. Evidence shows that adolescent mothers (15-19 years) are three times less likely to utilize ANC compared to adult mothers [15]. The uptake of ANC is influenced by socio-demographic and behavioural factors. Prior studies have reported that the uptake of ANC was associated with proximity to health facilities, maternal educational status, partner’s educational status, geographical region, access to health information and socioeconomic status [13, 14]. Though the World Health Organization recently recommended 8 or more ANC visits, adolescent and young mothers in Low- and Middle-Income Countries still do not obtain the previous recommendation of 4 or more ANC visits [16].

In Ghana, the maternal mortality ratio decreased from 398 deaths per 100,000 live births in 2003 to 308 deaths per 100,000 live births in 2017 [1]. This ratio is still high and above the Sustainable Development Goal 3 target. Adolescent pregnancy remains a public health concern in Ghana. For instance, about 109,888 adolescent pregnancies were recorded in 2020. Thus, 301 adolescent girls were impregnated every day or 13 adolescent girls were impregnated every hour [17]. The utilization of ANC among adolescent and young mothers in Ghana is below expectation. In addition, existing studies on ANC utilization focused on all pregnant women [18, 19], with little attention on adolescent and young mothers (10-24 years) who are at a higher risk of pregnancy-related complications. This is the maiden study to investigate the utilization of ANC among adolescent and young mothers in Ghana, using nationally representative data. Findings from this study would help inform maternal health policy and programming. Therefore, this study aimed to assess the prevalence of obtaining 4 or more ANC visits, and associated factors among adolescent and young mothers in Ghana, analyzing data from the 2017/18 Multiple Indicator Cluster Survey (MICS) [20].

## Methods

### Data source and study design

We analysed data from the sixth Multiple Indicator Cluster Survey in Ghana. The survey collected data on socio-demographic and health indicators at the national level. The target population for the survey were persons between the ages of 15-49 years, both males and females. A two-stage stratified sampling technique was employed to recruit participants into MICS. Participants were recruited across the former ten regions of Ghana using the 2010 Population and Housing Census as the sampling frame. In the first stage of the sampling, enumeration areas were selected from both rural and urban areas proportional to size. In the second stage, a systematic sampling technique was employed to select households within the enumeration areas. Regarding the women survey, data were collected from participants using Computer Assisted Personal Interviewing (CAPI).

The unweighted total sample size for the women survey (15-49 years) was 14,374 women. However, this study focused on mothers aged 15-24 years. Therefore, women aged 25-49 years (8538), women who did not utilize ANC services in the last 2 years to the survey (4707) and those who responded ‘do not know’ (7) were dropped/deleted before the analysis. The remaining sample for women aged 15-24 years was (unweighted) 1122 women. After adjusting for the sample weight, the sample size was reduced to (weighted) 947 women aged 15-24 years. Participation in MICS was voluntary and informed consent was obtained from adult participants (18-24 years) and caregivers of minors (15-17 years). Assent was obtained from minors. The 2017/18 MICS was approved by the Ghana Health Service Ethics Review Committee. Details about the 2017/18 MICS are provided elsewhere [20].

### Measurement

The main outcome of this study was ANC visits. This was a single continuous variable (i.e how many times you received antenatal care during this pregnancy?). During the analysis, the outcome was recoded into a dichotomous variable (1-3 ANC visits = 0 and 4 or more ANC visits = 1). The categorization was based on WHO’s previous recommendation of 4 or more ANC visits during pregnancy [13, 14]. Between 2002 and 2016, WHO advocated for a four-visit model of focused antenatal care, which prioritised the delivery of evidence-based interventions at each visit. The 2017/18 MICS was conducted barely a year after the WHO latest recommendation of 8 or more ANC visits. Hence, the implementation of the new policy was commencing in Ghana.

The following exposures were identified in the literature: mother’s age, mother’s educational status, marital status, household wealth index, area of residence, region [13, 16, 21] and health insurance membership [22–24]. Exposure to the media, including frequency of reading newspaper/magazine, frequency of listening to the radio and frequency of watching television, was also identified in the literature [25–27]. We also assessed the effect of mobile phone ownership and internet use on ANC utilization. Maternal age was coded as (15-19 years = 1 and 20-24 years = 2). Marital status was coded as (currently married/in union = 1, formerly married/in union = 2, and never married/in union = 3). Educational status was coded as (pre-primary or none = 0, primary = 1, junior high = 2, secondary = 3 and higher = 4). Household wealth index was coded as (poorest = 1, second = 2, middle = 3, fourth = 4 and richest = 5). In addition, area of residence was coded as (urban = 1 and rural = 2), while region was coded as (Western = 1, Central = 2, Greater Accra = 3, Volta = 4, Eastern = 5, Ashanti = 6, Brong- Ahafo = 7, Northern = 8, Upper East = 9 and Upper West =10). Items that measured exposure to the media included frequency of listening to the radio, watching television and reading newspaper/magazine, which were coded as (not at all = 0, less than once a week = 1, at least once a week = 2 and almost every day = 3). Mobile phone ownership was coded as (Yes = 1 and No = 2) and internet use was coded as (Yes = 1 and No = 2).

### Statistical analysis

Data were analysed with Stata/SE version 16 (StataCorp, College Station, Texas, USA). We employed both univariate and multivariable analyses. Descriptive statistics, including frequency, percentage and graphs, were computed at the univariate level. At the multivariable level, we computed Binary Logistic Regression. In the crude analysis, we assessed the effect of each exposure variable on the outcome variable. In the adjusted analysis, we assessed the combined effect of all the exposures on the outcome. To offset challenges associated with oversampling, we reported the weighted results. The adjusted model fitted reasonably well (*p* > 0.05) [28]. All statistical analyses were reported at the 0.05 significance level.

## Results

### Descriptive statistics

It was found that the majority (70%) of the participants were aged 20-24 years. About half (49%) of the participants had junior high school education and 61% were currently married/in a union. Exactly 9% of the participants were in the richest wealth quintile, while 26% were in the poorest wealth quintile. The majority of the participants resided in rural areas (65%) and 36% of them had enrolled on the National Health Insurance Scheme. Regarding exposure to the mass media, nine in ten participants did not read newspapers, 26% listen to the radio every day and 45% watched television every day. Also, more than half (90%) of the participants had never used the internet and more than five in ten participants owned a mobile phone. Details are provided in Table 1. Concerning ANC visits, the majority (84%) of the participants obtained 4 or more visits, while 16% obtained 1-3 ANC visits.

**Table 1:** Participants’ characteristics

Characteristic	Frequency	Percentage
***Age (years)***		
15-19	283	30
20-24	664	70
***Education***		
Pre-primary	113	12
Primary	230	24
Junior high	461	49
Senior high	135	14
Higher	9	1
***Marital status***		
Currently married/in union	573	61
Formerly married/in union	77	8
Never married/ in union	297	31
***Wealth index***		
Poorest	246	26
Second	226	24
Middle	222	23
Fourth	172	18
Richest	81	9
***Area of residence***		
Urban	328	35
Rural	619	65
*Region*		
Western	106	11
Central	116	12
Greater Accra	79	8
Volta	91	10
Eastern	148	15
Ashanti	190	20
Brong-Ahafo	77	8
Northern	81	9
Upper West	34	4
Upper East	25	3
***Health insurance status***		
Non-insured	606	64
Insured	341	36
***Frequency of reading newspaper***		
Not at all	890	94
Less than once a week	39	4
At least once a week	18	2
***Frequency of listening to the radio***		
Not at all	368	39
Less than once a week	159	17
At least once a week	173	18
Almost everyday	248	26
***Frequency of watching television***		
Not at all	295	31
Less than once a week	104	11
At least once a week	121	13
Almost everyday	428	45
***Ever used the internet***		
Yes	97	10
No	840	90
***Own a mobile phone***		
Yes	571	60
No	376	40

### Predictors of 4 or more ANC visits among adolescents and young mothers in Ghana

At the crude analysis level, it was found that mothers aged 20-24 years were 1.5 times (COR = 1.55; 95% CI: 1.00-2.38) more likely to obtain 4 or more ANC visits compared with those aged 15-19 years (reference category). Participants with senior high school education were 3 times (COR = 3.07; 95% CI: 1.13-8.33) more likely to obtain 4 or more ANC visits compared with those with pre-primary or no education (reference category). In addition, participants in the fourth wealth quintile (COR = 3.04; 95% CI: 1.39-6.66) had a higher likelihood of obtaining 4 or more ANC visits compared with those in the poorest wealth quintile (reference category). Participants residing in rural areas had decreased odds (COR = 0.51; 95% CI: 0.29-0.88) of obtaining 4 or more ANC visits compared with those residing in urban areas (reference category). Further, adolescents and young mothers who have ever used the internet were 6 times (COR = 6.72; 95% CI: 2.22-20.36) more likely to obtain 4 or more ANC visits compared with those who have never used the internet (reference category).

At the adjusted analysis level, the likelihood of obtaining 4 or more ANC visits was associated with higher educational status, socio-economic status, geographical region and exposure to the internet. For instance, adolescents and young mothers with junior high school education were 2 times (AOR = 2.19; 95% CI: 1.03-4.66) more likely to obtain 4 or more ANC visits compared with those with pre-primary or no education (reference category). Also, participants in the second wealth quintile were 2 times (AOR = 2.23; 95% CI: 1.20-4.16) more likely to obtain 4 or more ANC visits compared with those in the poorest wealth quintile (reference category). Further, adolescent and young mothers residing in the Upper East region were 6 times (AOR = 6.30; 95% CI: 1.03-38.74) more likely to obtain 4 or more ANC visits compared with those in the Greater Accra region (reference category). Adolescents and young mothers who had ever used the internet were 4 times (AOR = 4.38; 95% CI: 1.09-17.64) more likely to obtain 4 or more ANC visits compared with those who had never used (reference category) Table 2.

**Table 2:** Regression analysis of ANC visits among adolescent and young mothers in Ghana

Exposures	Crude Odd Ratio (95% CI)	Adjusted Odd Ratio (95% CI)
***Age (years)***		
15-19	1(reference category)	1(reference category)
20-24	1.55 (1.00-2.38) *	1.34 (0.81-2.20)
***Education***		
Pre-primary	1(reference category	1(reference category)
Primary	1.15 (0.54-2.46)	1.76 (0.79-3.86)
Junior high	1.59 (0.83-3.04)	2.19 (1.03-4.66) *
Senior high	3.07 (1.13-8.33) *	2.40 (0.74-7.78)
***Marital status***		
Currently married/in union	1(reference category	1(reference category)
Formerly married/in union	1.47 (0.61-3.52)	1.53 (0.54-4.29)
Never married/ in union	0.72 (0.46-1.13)	0.74 (0.44-1.25)
***Wealth index***		
Poorest	1(reference category	1(reference category)
Second	1.86 (1.08-3.19) *	2.23 (1.20-4.16) *
Middle	1.04 (0.57-1.92)	1.01 (0.50-2.04)
Fourth	3.04 (1.39-6.66) *	2.32 (0.88-6.17)
Richest	2.71 (0.60-12.14)	1.69 (0.27-10.56)
***Areas of residence***		
Urban	1(reference category	1(reference category)
Rural	0.51 (0.29-0.88) *	0.73 (0.38-1.42)
*Region*		
Greater Accra	1(reference category	1(reference category)
Western	0.79 (0.26-2.94)	1.26 (0.28-5.75)
Central	0.80 (0.26-2.47)	1.05 (0.28-4.03)
Volta	0.27 (0.09-0.83) *	0.45 (0.11-1.77)
Eastern	0.39 (0.13-1.15)	0.54 (0.15-1.88)
Ashanti	0.75 (0.25-2.26)	1.10 (0.31-3.90)
Brong-Ahafo	0.53 (0.17-1.59)	0.85 (0.22-3.22)
Northern	0.83 (0.26-2.66)	2.00 (0.48-8.35)
Upper West	0.55 (0.19-0.60)	1.25 (0.32-4.91)
Upper East	2.56 (0.54-12.27)	6.30 (1.03-38.74) *
***Health insurance status***		
Non-insured	1(reference category	1(reference category)
Insured	1.32 (0.81-2.16)	0.85 (0.51-1.39)
***Frequency of reading newspaper***		
Not at all	1(reference category	1(reference category)
Less than once a week	2.20 (0.73-6.64)	0.72 (0.22-2.38)
At least once a week	1.70 (0.27-10.69)	0.43 (0.06-2.87)
***Frequency of listening to the radio***		
Not at all	1(reference category	1(reference category)
Less than once a week	1.50 (0.74-3.04)	1.34 (0.66-2.72)
At least once a week	1.43 (0.76-2.72)	1.43 (0.72-2.85)
Almost everyday	1.50 (0.81-2.81)	1.41 (0.76-2.59)
***Frequency of watching television***		
Not at all	1(reference category	1(reference category)
Less than once a week	0.98 (0.44-2.19)	0.86 (0.39-1.90)
At least once a week	1.40 (0.70-2.79)	1.67 (0.73-3.85)
Almost everyday	1.36 (0.77-2.39)	0.93 (0.51-1.71)
***Ever used the internet***		
No	1(reference category	1(reference category)
Yes	6.72 (2.22-20.36) *	4.38 (1.09-17.64) *
***Own a mobile phone***		
No	1(reference category	1(reference category)
Yes	1.37 (0.91-2.08)	0.90 (0.55-1.47)

* *p*-value < 0.05

## Discussion

It was revealed that the majority (84%) of the mothers obtained the recommended ANC visits. This national-level prevalence is similar to findings at the district level. For instance, a study in the Yendi Municipality found a prevalence of 83.9% among adolescent mothers [14]. However, the prevalence in this study is higher than findings in Nigeria (35.1%) [29], Bangladesh (30%) [30] and India (22.9%) [31]. The differences in findings can be attributed to the implementation of the Free Maternal Health Care Policy (FMHCP) by the government of Ghana in 2008. With this policy, pregnant women who enrol on the National Health Insurance Scheme (NHIS) have access to free maternal healthcare services, including ANC services.

On the other hand, a substantial proportion (16%) of adolescent and young mothers did not obtain the recommended ANC visits. This is likely to derail efforts towards achieving SDG 3 since optimal ANC utilization is crucial for reducing maternal mortality [4]. The prevalence of underutilization of ANC (16%) is higher than findings in developed countries (5%) [7]. This may be attributed to differences in contextual factors, such as socio-cultural norms and health system factors. For instance, adolescents may delay in accessing ANC services due to fear of stigma or being expelled from school [32]. Also, negative attitudes of health providers towards adolescent mothers coupled with distance to health facilities might have accounted for the differences in the findings [14, 33].

The salient factors associated with obtaining the recommended ANC visits were higher educational status, higher socio-economic status, exposure to the internet, and geographical region. It was revealed that adolescent and young mothers who had junior high school education were more likely to obtain 4 or more ANC visits. This finding is consistent with prior studies in low-and middle-income countries [16, 29]. For instance, a study revealed that Indonesian adolescent mothers with higher education were more likely to utilize ANC services compared with those with lower education [34]. Also, a systematic review of studies from sub-Saharan Africa showed that women with higher education were more likely to obtain the recommended ANC visits [35]. Educated mothers have more access to health information, appreciate the causes of adverse pregnancy outcomes and the importance of ANC to the wellbeing of the mother and the unborn baby [36]. In addition, educated mothers have greater autonomy to make decisions and financial access to quality healthcare [37].

In addition, it was revealed that adolescent and young mothers in the second wealth quintile were more likely to obtain the recommended ANC visits. This finding is consistent with previous studies in developing countries where economic inequities were observed in maternal healthcare service utilization [34]. Evidence shows that socio-economic status significantly affects ANC utilization among adolescent mothers [15, 16, 21, 29, 32]. This finding is understandable because young mothers from poor households are less likely to have financial access to maternal healthcare compared with those from wealthy households [31]. A previous study reported that pregnant women in Ghana still pay for some maternal health services such as drugs, urine and blood tests and ultrasound scans despite the Free Maternal Healthcare Policy [38].

Internet use was also associated with optimal ANC utilization. This finding is consistent with previous studies in Malawi where women who received family planning messages through the internet had higher odds of antenatal care utilisation [39]. Evidence shows that women access pregnancy-related information on the internet, which has the potential to influence their health-seeking behaviours [40]. The geographical region was another salient factor associated with ANC utilization [16]. Adolescent and young mothers in the Upper East region were more likely to obtain the recommended ANC visits. The Upper East region has the highest ANC coverage in Ghana [41], hence this finding is understandable.

### Implications and recommendations of findings

The findings of this study provide relevant information for maternal health policy and programming. For instance, a substantial proportion of adolescent and young mothers did not obtain the recommended ANC visits. These young mothers are at a higher risk of pregnancy and childbirth complications as well as negative birth outcomes. This may delay the achieving of Sustainable Development Goals 3, which aims to ensure healthy lives and promote wellbeing at all ages, including adolescent and young mothers. It is, therefore, necessary for stakeholders, including the Ministry of Health and Ghana Health Services, to invest resources that will help increase ANC coverage among young mothers. Stakeholders can leverage existing youth-friendly initiatives such as the Adolescent Health and Development (ADHD) programme. Currently, the ADHD programme does not cover ANC services, hence, stakeholders should consider incorporating these services. Also, the Ghana Health Service should consider separating young mothers from adult mothers during ANC visits to help encourage ANC utilization among adolescent mothers. Owolabi and colleagues [4] revealed that adult mothers stigmatize pregnant adolescent girls. Moreover, stakeholders should strengthen efforts towards providing Focused Antenatal Care. This may help increase ANC utilization among adolescent and young mothers in Ghana.

Further, adolescent and young mothers from poor households, those with lower education and no internet exposure were less likely to obtain optimal ANC visits. This suggests that poor mothers still face financial barriers to accessing ANC services despite the Free Maternal Health Care Policy. This has the potential to delay progress towards improving maternal and child health outcomes as well as achieving SDGs 3. Therefore, the National Health Insurance Authority needs to liaise with health service providers to eliminate all forms of unapproved charges (fees for medication, ultrasound scan, urine and blood test) on maternal healthcare services as reported by Ziblim and colleagues [14] in Northern Ghana. In addition, stakeholders must invest resources in promoting girl child education. Hence, Ghana’s free basic and secondary education policy is commendable. The internet may provide a golden opportunity for stakeholders to increase ANC coverage among young mothers. With the rapid increase in internet penetration in the country, stakeholders can leverage the internet to raise awareness and educate adolescent and young mothers about the importance of ANC and danger signs in pregnancy and childbirth.

### Strength and limitations

This is the maiden study in Ghana to estimate the prevalence of ANC visits among adolescent and young mothers using national representative data. Although this study provides invaluable information for maternal health policy, it is not devoid of limitations. This study focused on socio-demographic factors and ANC utilization; hence the interpretation of the findings must be done with caution. Another limitation of this study is the small sample size. Also, quantitative surveys are unable to expose the many intricate views of participants regarding a subject matter. Future studies should, therefore, consider adopting qualitative designs as well as assessing more exposures. For instance, the 2017/18 MICS did not collect data on some potential exposures of ANC utilization among adolescent and young mothers. These exposures include school attendance, pregnancy history and violence against women, hence they should be included in future surveys.

## Conclusion

This study showed high utilization of ANC services among adolescent and young mothers. Optimal utilization of ANC was influenced by higher educational status, socio-economic status, exposure to the internet and residing in the Upper East region. Efforts to increase ANC coverage among adolescent and young mothers should focus on promoting girl child education and removing financial barriers to accessing healthcare. Going forward, stakeholders must focus on addressing socio-economic inequalities as part of efforts to improve maternal and child health indicators.

## Data Availability

The data used in this study is owned by UNICEF, therefore, the authors cannot share the data. The datasets generated and/or analysed during the current study are available in the UNICEF repository. Interested persons can contact UNICEF for the data via accra@unicef.org. The authors confirm they did not have any special access or privileges to the data that other researchers would not have.
